# A Critical Analysis of All-Cause Deaths during COVID-19 Vaccination in an Italian Province

**DOI:** 10.3390/microorganisms12071343

**Published:** 2024-06-30

**Authors:** Marco Alessandria, Giovanni M. Malatesta, Franco Berrino, Alberto Donzelli

**Affiliations:** 1 Department of Life Sciences and Systems Biology, University of Turin, 10123 Turin, Italy; marco.alessandria@unito.it; 2 Scientific Committee of the Foundation “Allineare Sanità e Salute”, 51100 Pistoia, Italy; info@villapacinotti.it; 3 Department of Predictive and Preventive Medicine, Fondazione IRCCS Istituto Nazionale dei Tumori, 20133 Milan, Italy; francoberrino@gmail.com; 4 Independent Medical-Scientific Commission, Foundation “Allineare Sanità e Salute”, 20131 Milan, Italy

**Keywords:** pandemic vaccine effectiveness, immortal time bias, healthy-vaccinee bias, total mortality

## Abstract

Immortal time bias (ITB) is common in cohort studies and distorts the association estimates between the treated and untreated. We used data from an Italian study on COVID-19 vaccine effectiveness, with a large cohort, long follow-up, and adjustment for confounding factors, affected by ITB, with the aim to verify the real impact of the vaccination campaign by comparing the risk of all-cause death between the vaccinated population and the unvaccinated population. We aligned all subjects on a single index date and considered the “all-cause deaths” outcome to compare the survival distributions of the unvaccinated group versus various vaccination statuses. The all-cause-death hazard ratios in univariate analysis for vaccinated people with 1, 2, and 3/4 doses versus unvaccinated people were 0.88, 1.23, and 1.21, respectively. The multivariate values were 2.40, 1.98, and 0.99. Possible explanations of this trend of the hazard ratios as vaccinations increase could be a harvesting effect; a calendar-time bias, accounting for seasonality and pandemic waves; a case-counting window bias; a healthy-vaccinee bias; or some combination of these factors. With 2 and even with 3/4 doses, the calculated Restricted Mean Survival Time and Restricted Mean Time Lost have shown a small but significant downside for the vaccinated populations.

## 1. Introduction

The SARS-CoV-2 pandemic has led to an unprecedented effort to generate real evidence on the safety and effectiveness of various treatments, mRNA vaccines included. Information from similar studies is crucial for government leaders and policymakers to evaluate their health crisis policies. Therefore, it is essential that such studies are truly reliable and as free as possible from bias. Unfortunately, as already observed in articles published during the pandemic [1,2], in observational cohort studies, incorrect management of follow-up times may introduce the so-called immortal time bias (ITB) in favor of the exposed group (for studies on mRNA vaccines, the exposed are vaccinated people). This systematic error can bias estimates of measures of association and generate misleading results.

Nevertheless, ITB still remains present in several cohort studies. As highlighted in a recently published paper [3], a study on the safety of COVID-19 vaccines in the population of an Italian province is no exception [4]. A possible explanation for why ITB is still largely prevalent in such cohort studies may be that the structure of ITB is still poorly understood [2]. 

Furthermore, recent articles [5,6,7] have highlighted that observational studies on the effectiveness of COVID-19 vaccines are subject to inherent biases, including differences in testing strategies and disparities in hospitalizations between vaccinated and non-vaccinated people or the doubtful attribution of causes of death between vaccinated and unvaccinated people. In particular, the incorrect classification of deaths is a critical factor for the evaluation of vaccine effectiveness (VE) concerning fatal outcomes. Therefore, “all-cause deaths” seems to be the outcome least affected by misclassification.

A recent article [8] highlighted the persistence of significant excesses of all-cause deaths in the years 2021 and 2022, following the onset of the pandemic, despite the containment measures and vaccination programs. However, without distinguishing deaths based on vaccination status, it is impossible to establish whether there is a link between these excess deaths and COVID-19 mass vaccinations. 

Therefore, there is a crucial need for studies that relate all-cause deaths to the vaccination status of large populations with a long follow-up, adjusted for age, gender, and confounding factors such as previous morbidities. We welcomed with great interest the paper by Rosso et al. [9] on the effectiveness of COVID-19 vaccines in the general population of the Italian province of Pescara, divided by vaccination status, with a follow-up of two years. This interest lies in the size of the population analyzed, in the long follow-up, and in also considering the outcomes of all-cause death and previous comorbidity as confounding factors. Moreover, this new study showed that people receiving only one or two vaccine doses had a significantly higher risk of all-cause death (HRs 1.40 and 1.36, respectively; both *p* < 0.001), while the subjects receiving three or more vaccine doses showed a substantially lower risk of death (HR 0.22; 95% CI: 0.20–0.23). Unfortunately, this study is also affected by ITB, as well as the previous study by the same authors cited above [4]. Considering the interest in the data underlying this study, we asked the authors for the original dataset on which the study was based, and they kindly provided it to us.

Considering that overall COVID-19-related deaths represent a minority portion of total deaths, equal to 9,0% in Italy according to the latest available data from ISTAT in the year 2021 [10], even assuming that vaccination can lead to very high reductions in the risk of COVID-19-related deaths [11], this will affect the risk of all-cause deaths only marginally. Therefore, we hypothesize that the correction of ITB can push the hazard ratio estimates for the “all-cause deaths” outcome towards unity, or at least to a limited effect, differently from the result obtained by Rosso et al. [9].

This retrospective cohort study aimed to correct ITB in order to verify the real impact of the vaccination campaign by comparing the risk of all-cause death between the vaccinated population and the unvaccinated population of the province of Pescara (Italy).

## 2. Materials and Methods

This retrospective cohort study used information collected from the dataset kindly provided by Rosso et al. [9]. The dataset was restructured in order to correct the ITB recognized by Berrino et al. [3] in a previous paper by the same authors using almost the same dataset [4]. This research was carried out following the rules of the Declaration of Helsinki for the use of data.

### 2.1. Data Sources

All the information contained in the dataset provided by Rosso et al. and used for this analysis was extracted from the Italian National Healthcare System. The population considered is that of residents or those domiciled in the province of Pescara on 1 January 2021, aged 10 years and older, without a positive SARS-CoV-2 swab at the date of the follow-up start. 

Vaccination data were acquired from the official regional SARS-CoV-2 vaccination dataset, up to 31 December 2022. 

The follow-up considered ranges between 1 January 2021 and 15 February 2023. 

The main confounders used to verify the association between exposure (vaccination statuses) and outcome (all-cause deaths) were identified using the co-pay exemption database and administrative discharge abstracts from the last ten years to extract the following conditions for each individual, and all the information was merged through encrypted fiscal code [9]. Additional information collected to adjust the estimated HRs included nine covariates: sex, age, hypertension, diabetes, chronic obstructive pulmonary disease (COPD), cardiovascular disease (CVD), kidney diseases, cancer, and infection (individuals infected by SARS-CoV-2).

### 2.2. Immortal Time Bias Correction 

To correct ITB we aligned the entire population on a single index date (1 January 2021), calculating the time spent unvaccinated for the 1-dose population, the time spent unvaccinated and with 1-dose for the 2-dose population, and, finally, the time spent unvaccinated and with 1-dose and 2–doses for the 3/4-dose population. 

The time spent by each individual in their respective cohorts was calculated as person-days according to the following formula:

*person*−*days* = Σ*fwi*

where *fwi* is the follow-up of the *i-th* subject of the cohort. 

In this way, the cohort was divided as follows:

(a) “Unvaccinated”: in this group, we included never-vaccinated individuals, and vaccinated individuals before receiving 1 or more doses;
(b) “1–dose”: in this group, we included all individuals vaccinated with 1 dose and all individuals vaccinated before they received 2 or more doses;
(c) “2–doses”: in this group, we included all individuals vaccinated with 2 doses and all vaccinated individuals before they received 3 or more doses;
(d) “3/4 doses”: in this group, we included all vaccinated individuals with 3 or more doses.

Accordingly, the follow-up periods were as follows:

(a) “Unvaccinated”: the follow-up started on 1 January 2021 and ended on the day of death, or of the 1st dose, or on 15 February 2023;
(b) “1–dose”: the follow-up started on the 15th day after the 1st dose and ended on the day of death, or of the 2nd dose, or on 15 February 2023;
(c) “2–doses”: the follow-up started on the 15th day after the 2nd dose and ended on the day of death, or of the 3rd dose, or on 15 February 2023;
(d) “3/4 doses”: the follow-up started on the 15th day after the 3rd dose and ended on the day of death or on 15 February 2023.

### 2.3. Follow-Up 

The entire period of observation lasted 775 days considering the start and end dates indicated above (1 January 2021 and 15 February 2023). The average follow-up of each cohort obtained from the restructuring of the dataset are as follows:

(a) “Unvaccinated”: 258 days;
(b) “1 dose”: 61 days;
(c) “2 doses”: 247 days;
(d) “3/4 doses”: 400 days.

### 2.4. Outcome 

Two outcomes were considered in this study:

(a) “All-cause deaths”;
(b) “COVID-19-related deaths”.

### 2.5. Statistical Analysis 

Cox proportional hazard analysis was used to compute the adjusted hazard ratio (HR) of all covariates used in the model. In order to compare survival distributions between the unvaccinated group and the several vaccination statuses considered, Log-rank test was used and α level was fixed to 0.05. The exposure time was defined in days. Mean and Standard deviation were used to describe measures of central tendency and variability of the continuous variables.

We considered “All-cause deaths” as the dependent variable in the model, and “Groups” as the independent variable that compares the unvaccinated population and “1-dose” population or “2-doses” population or “3/4 doses” population. 

With the aim to verify the validity of assumption of the model, Schoenfeld’s test was used considering both the value of the global test and each covariate, so we can use the appropriate stratification if the assumptions of the model were not satisfied. In particular, in the multivariable analysis, the HRs were obtained by a stratification of the covariates which were significant to the global Schoenfeld’s test. Furthermore, we plotted log cumulative hazard in order to ensure graphically the validity of the assumptions of the model. 

For covariates, the HRs must be interpreted by comparing the population with the disease to the population without the disease at the same vaccine dose. 

Finally, we used Restricted Mean Survival Time (RMST) and Restricted Mean Time Lost (RMTL) to estimate the difference and the ratio between groups, inasmuch as possible as the construction of a time-dependent variable did not allow us to correct the model assumptions for the 2-dose and 3-dose vaccination status. RMST is the best index of “life expectancy” in those comparison where the assumptions of the model were not respected [12], while RMTL can approximate the HR in the absence of proportional hazard assumption [13]. The truncation time, tau (τ), was fixed to be equal to the minimum of the largest observed times of each of the two groups. 

Data were processed using R studio (version 2023.09.0).

## 3. Results

### 3.1. Population Distribution after ITB Correction

At the time of the index date considered for the alignment of follow-ups, the unvaccinated subjects were the entire population (290,727 subjects) with an average age of 48.9 ± 20.8 years and 48.8% were male. With the start of the administration of the first doses, the overall population was represented by 245,741 subjects with an average age of 49.7 ± 20.7 years and 48.3% were male. The population that completed the vaccination cycle with the second dose was represented by 234,287 subjects with an average age of 50.1 ± 20.7 years and 48.1% were male. Finally, the booster doses (3/4 doses) were administered to an overall population of 186,684 subjects with an average age of 52.5 ± 20.2 years and 47.8% were male.

The distribution of risk factors and comorbidities remains almost constant among the populations considered due to their intrinsic dynamic characteristics.

All these demographic characteristics and the comorbidity are summarized in Table 1.

### 3.2. COVID-19-Related Death Classifications

The analysis of the classification of “COVID-19-related deaths” showed non-negligible percentages of deaths without severe COVID-19 disease and deaths due to COVID-19 over 90 days from the last swab, as well as a small percentage of COVID-19-related deaths in the same day of the last swab. All percentages are summarized in Table 2.

### 3.3. One Dose versus Unvaccinated

In the Log-rank test of the univariate analysis, the one-dose population shows an HR of 0.88 (CI_95_ = 0.78–1.00) versus the unvaccinated; the hypertensive population shows an HR of 12.59 (CI_95_ = 11.58–13.69) compared to the non-hypertensive population; the diabetic population shows an HR of 8.07 (CI_95_ = 7.31–8.90) compared to the non-diabetic population; the CVD population shows an HR of 11.56 (CI_95_ = 10.63–12.57) compared to the non-CVD population; the population with kidney disease shows an HR of 17.89 (CI_95_ = 16.08–19.90;) compared to the population with no kidney disease; the cancer population shows an HR of 9.34 (CI_95_ = 8.51–10.25); the SARS-CoV-2 infected population shows an HR of 0.58 (CI_95_ = 0.53–0.63) compared to the population without it; the HR of the covariate age is 1.11 (CI_95_ = 1.11–1.11); the male population shows an HR of 0.87 (CI_95_ = 0.81–0.95) compared the female population; and, finally, the COPD population shows an HR of 7.11 (CI_95_ = 6.40–7.91) compared to the non-COPD population (Table 3).

The Log-rank test of the multivariate analysis gave the following results: the HR for “All-cause deaths” between one dose versus unvaccinated is 2.40 (CI_95_ = 2.00–2.88). The hypertensive population shows an HR of 1.49 (CI_95_ = 1.23–1.82) compared to the non-hypertensive population; the diabetic population shows an HR of 2.00 (CI_95_ = 1.60–2.49) compared to the non-diabetic population; the CVD population shows an HR of 1.60 (CI_95_ = 1.31–1.96) compared to the non-CVD population; the population with kidney disease shows an HR of 1.77 (CI_95_ = 1.35–2.34) compared to the population with no kidney disease; the male population shows an HR of 1.50 (CI_95_ = 1.27–1.78) compared to the female population; and, finally, the COPD population shows an HR of 2.01 (CI_95_ = 1.56–2.60) compared to the non-COPD population (Table 3).

### 3.4. Two Doses versus Unvaccinated

Through the Log-rank test of the univariate analysis, the two-dose population shows an HR of 1.23 (CI_95_ = 1.16–1.32) versus that of the unvaccinated people; the hypertensive population shows an HR of 11.47 (CI_95_ = 10.76–12.23) compared to the non-hypertensive population; the diabetic population shows an HR of 6.71 (CI_95_ = 6.23–7.23) compared to the non-diabetic population; the CVD population shows an HR of 10.88 (CI_95_ = 10.21–11.60) compared to the non-CVD population; the population with kidney disease shows an HR of 16.83 (CI_95_ = 15.56–18.20) compared to the population with no kidney disease; the cancer population shows an HR of 8.65 (CI_95_ = 8.07–8.27); tThe SARS-CoV-2 infected population shows an HR of 0.35 (CI_95_ = 0.32–0.38) compared to the population without it; the covariate age shows an HR of 1.11 (CI_95_ = 1.11–1.12); the male population shows an HR of 0.95 (CI_95_ = 0.89–1.01); and the COPD population shows an HR of 6.28 (CI_95_ = 5.79–6.82) compared to the non-COPD population (Table 3).

In the multivariable analysis, the HR for “All-cause deaths” between two doses versus unvaccinated is 1.98 (CI_95_ = 1.75–2.24). The diabetic population shows an HR of 1.74 (CI_95_ = 1.38–2.20) compared to the non-diabetic population; the CVD population shows an HR of 1.78 (CI_95_ = 1.44–2.20) compared to the non-CVD population; the population with kidney disease shows an HR of 2.44 (CI_95_ = 1.84–3.24) compared to the population with no kidney disease; and the COPD population shows an HR of 2.89 (CI_95_ = 2.18–3.84) compared to the non-COPD population (Table 3).

Restricted Mean Survival Time for the two-dose population (τ = 739 days) is 728.9 (CI_95_ = 728.3–729.5) days compared to the unvaccinated population which is 731.6 (CI_95_ = 731.3–731.9), while the between-group contrast is −2.7 days (CI_95_= −3.4–−2.0). The Restricted Mean Time Lost ratio is 1.37 (CI_95_ = 1.27–1.48) (Table 4).

### 3.5. 3/4 Doses versus Unvaccinated

Through the Log-rank test of the univariate analysis, the 3/4-dose population shows an HR of 1.21 (CI_95_ = 1.14–1.29) versus that of the unvaccinated; the hypertensive population shows an HR of 9.65 (CI_95_ = 9.09–10.24) compared to the non-hypertensive population; the diabetic population shows an HR of 5.90 (CI_95_ = 5.51–6.31) compared to the non-diabetic population; the CVD population shows an HR of 10.03 (CI_95_ = 9.45–10.63) compared to the non-CVD population; the population with kidney disease shows an HR of 15.89 (CI_95_ = 14.78–17.08) compared to the population with no kidney disease; the cancer population shows an HR of 7.62 (CI_95_ = 7.15–8.12); The SARS-CoV-2 infected population shows an HR of 0.61 (CI_95_ = 0.58–0.66) compared to the population without it; the HR of the covariate age is 1.12 (CI_95_ = 1.11–1.12); the male population show an HR of 0.98 (CI95 = 0.93–1.04) compared to the female population; and finally the COPD population shows an HR of 5.96 (CI_95_ = 5.52–6.43) compared to the non-COPD population (Table 3).

In the multivariable analysis, the HR for “All-cause deaths” between 3/4 doses versus that of the unvaccinated is 0.99 (CI_95_ = 0.90–1.09). The hypertensive population shows an HR of 1.24 (CI_95_ = 1.11–1.39) compared to the non-hypertensive population; the diabetic population shows an HR of 1.68 (CI_95_ = 1.48–1.90) compared to the non-diabetic population; the CVD population shows an HR of 1.86 (CI_95_ = 1.65–2.09) compared to the non-CVD population; the population with kidney disease shows an HR of 2.47 (CI_95_ = 2.11–2.89) compared to the population with no kidney disease; the male population shows an HR of 1.37 (CI_95_ = 1.24–1.51) compared to the female population; and, finally, the COPD population shows an HR of 1.85 (CI_95_ = 1.59–2.15) compared to the non-COPD population (Table 3).

Restricted Mean Survival Time for the three-dose population (τ = 579 days) is 573.7 (CI_95_ = 573.5–573.9) days compared to the unvaccinated population which is 574.4 (CI_95_ = 574.2–574.7), while the between-group contrast is −0.8 days (CI_95_ = −1.1–−0.5). The Restricted Mean Time Lost ratio is 1.17 (CI_95_ = 1.10–1.24) (Table 5).

## 4. Discussion

This retrospective cohort study aims to correct for the ITB from the dataset kindly provided by Rosso et al. [9], which refers to the population of the province of Pescara, in order to verify the real impact of the vaccination campaign by comparing the survival curves for the “all-cause deaths” outcome between the cohorts of people vaccinated with 1, 2, and 3/4 doses and the cohort of unvaccinated people. 

The hypotheses that we advanced following the ITB correction concerned a possible shift in the HR estimates towards unity compared to those obtained by Rosso et al. [9]. These assumptions did not meet the comparisons between the unvaccinated population and those with 1 and 2 doses, while they were confirmed for the comparison with the population with 3/4 doses. Specifically, we obtained point estimates of adjusted HRs that were higher than those obtained by Rosso et al. as follows: 2.40 versus 1.40 in the comparison between the 1-dose population and the unvaccinated population, 1.98 versus 1.36 in the comparison between the 2-dose population and the unvaccinated population, and 0.99 versus 0.22 in the comparison between the 3/4-dose population and the unvaccinated population.

In our analyses, we focused only on the all-cause-death outcome, and we decided not to consider the “COVID-19-related deaths” outcome. 

The reason for this choice is that we found specific indications of unreliability in the attributions of deaths to COVID-19 during the exploration of the dataset. First, COVID-19-related deaths appear greatly overestimated. Indeed, in the dataset provided by Rosso et al., COVID-19 deaths represent 22.3% of deaths from all-causes (see Table 2), more than twice the amount of the ISTAT data for the province of Pescara (the last update is for the year 2021, with a total of 10% COVID-19-related deaths; n = 398/3978) and 2.5 times higher than the national one, i.e., 9.0%% [10]. Second, the “COVID-19-related death” outcome presented some questionable attributions, which can be summarized as follows: COVID-19-related death without severe COVID-19, COVID-19-related death on the same day as the positive swab, and COVID-19-related death occurring more than 90 days after the last positive swab (Table 2). In several cases, the first two attributions can find some reasonable explanations, e.g., deaths without COVID-19 severe disease (classified using hospitalization as a proxy for severity) occurred, especially among elderly people cared for in nursing homes or by primary care services, or diagnoses made for people brought to the emergency departments with very severe symptoms and who died the same day. On the contrary, the third, which involves over 30% of deaths related to COVID-19, appears very difficult to justify. However, given the strong overestimation of deaths related to COVID-19 reported above, incorrect classification was also likely part of the other two attributions.

Therefore, a reliable estimate of the statistical parameters for this outcome is impossible, and the choice of disregarding COVID-19-related deaths can be valid.

The results obtained from our analyses of all-cause-death outcome are a consequence of the alignment carried out to correct ITB, which led to a different population composition compared to the article by Rosso et al. [9]. In fact, in the unvaccinated population, we inserted never-vaccinated and not-vaccinated individuals prior to receiving one or more doses; in the one-dose population group were inserted all individuals vaccinated with one dose and all individuals vaccinated prior to receiving two or more doses; in the two-dose population were inserted all individuals vaccinated with two doses and all individuals vaccinated prior to receiving two or more doses; and, finally, in the three- or four-dose population, we inserted all individuals vaccinated with three or more doses.

To obtain as complete a picture as possible of the relationships between the unvaccinated and vaccinated populations, we included the following:

− Univariate and multivariate analysis, to highlight the differences between the adjusted and unadjusted data.
− The HRs of the covariates, because they represent the comparison between subjects who have a specific comorbidity with subjects who do not have it, for the same vaccination doses. This may allow for appropriate public health assessments and reassessments of the opportunity to reserve vaccination priority for specific categories of frail subjects.
− RMST and RMTL, as they provide information on the loss of life expectancy among the compared populations and can replace the HRs when the model assumptions are not met despite the corrections made.

The univariate analysis, carried out using the Cox proportional hazard model, shows an increase in the risk of the vaccinated compared to in the unvaccinated, as one moves from the first to the subsequent doses. This confirms what has already been highlighted in our previous article [4]. Accordingly, the HR was slightly lower than 1 with the first dose, while with the second and third doses, the risk for the vaccinated resulted in being significantly higher (more than 20%) than that of the unvaccinated.

However, age, sex, and previous pathologies are confounding factors affecting the HR of all-cause deaths of the vaccinated compared to the unvaccinated. Therefore, only a multivariate analysis allows for a more reliable estimate of the HRs for the different vaccination statuses compared to the unvaccinated.

The HRs obtained in the multivariable analysis represent the best fit of the model achieved by stratification of the covariates of hypertension, cancer, infection, sex, and age. Despite this procedure, the global Schoenfeld’s test remained significant for the comparison between the 2-dose and 3/4-dose population with the unvaccinated population whereby the estimate of the HRs could be inaccurate. For this reason, we calculated the RMST difference that represents the best index of “life expectancy” in those comparisons where the assumptions of the model were not met [12], and with the RMTL ratio that can approximate the HR in the absence of proportional hazard assumption [13]. The RMST difference represents the days of life lost by the vaccinated population compared to those of the unvaccinated one, while the RMTL ratio represents the percentage of life expectancy lost in the vaccinated population compared to in the unvaccinated one. The differences in RMSTs between the vaccinated and unvaccinated are significant for both the two-dose and the three-or-more-dose groups. They may appear irrelevant (in the order of a few days), but they refer to a limited period of time (739 days for those vaccinated with two doses and 579 days for those vaccinated with three or more doses). They could be compared with the entire life expectancy of an individual, which in the province of Pescara has an average value of 82.6 years [14] (corresponding to 30,149 days).

For those vaccinated with two doses, the loss of life expectancy (RMTL) in 739 days is 1.37 (CI 95 = 1.27–1.48; *p* < 0.0001) times that of the unvaccinated. This means that the subjects vaccinated with two doses lost 37% of life expectancy compared to the unvaccinated population during the follow-up considered. The difference between the life expectancy (RMST) of the vaccinated and that of the unvaccinated limited to the period considered is −2.71 (CI 95 = −3.40 to −2.01; *p* < 0.0001) days. However, to have an easily understandable comparison, if we extrapolate this result to the entire life expectancy of the Pescara population, we will obtain a loss of life expectancy difference of about −3.6 months. Obviously, this is an extrapolation made for the sole purpose of giving the reader an idea of the order of magnitude of the RMTL. It may not constitute a realistic prediction, as it would presuppose health conditions to be invariant over time, an assumption that is very difficult to realize.

For those vaccinated with three or more doses, the RMTL in 579 days is 1.17 times (CI 95 = 1.10–1.24; *p* < 0.0001) the one of the unvaccinated. The difference between the vaccinated and unvaccinated RMST in the considered period is 0.764 days (CI 95 = −1.07 to −0.46; *p* < 0.0001). With the above extrapolation, it would correspond to a loss of life expectancy of −1.31 months.

For comparison, between 2019 and 2022, the life expectancy in the province of Pescara fell by 1.0 year, from 83.6 to 82.6 years [14], indeed corresponding to an annual loss of 4.0 months.

There are several possible interpretations of these results. Rosso et al. [9] hypothesize that, as a consequence of the green-pass policies, the sickest subjects were concentrated among the vaccinated with one and two doses. However, the figures in the dataset do not support this hypothesis. Indeed, the population of those vaccinated with three or more doses displays the highest percentage of subjects with pathologies (28% versus 16% of those vaccinated with only two doses, and 17% of those with only one dose). Moreover, in Italy the release of exemptions was very limited, while the subjects with pathologies were vaccinated with a priority indication and were induced to continuously be vaccinated with one or two booster doses.

Among the possible explanations of the HR trend as vaccinations increase, one must consider the so-called *harvesting effect*, due not only to the deaths of the elderly and frail subjects, who were vaccinated with priority, but also likely to vaccine adverse effects, including some fatal outcomes. Indeed, if for the individuals vaccinated with three or more doses the HR shows no effect on all-cause deaths, assuming that vaccination against COVID-19 can reduce COVID-19-related deaths, we can assume that this reduction is counterbalanced by an increase in deaths from other causes. Therefore, we should admit that vaccination increases the risk of death from causes other than COVID-19, or by direct damage (adverse effects), or by indirect damage, e.g., to the immune system [15,16,17]. Therefore, it might be that the risk of death is greater for one dose than for two, and greater for two than for three doses, because individuals more liable to harm are already dead after the first and the second doses. So, it will be important to continue the follow-up of the cohort, to capture any long-term damage. To understand the impact of the harvesting effect, deaths among those vaccinated with one or two doses represent about half of all deaths that occurred among the vaccinated (47.2%, the majority of them among those vaccinated with two doses) in the two-year follow-up.

Another specific explanation could lie in the so-called calendar-time bias: it consists of not taking into account either seasonality [18] or pandemic waves. In this present study, such a bias may have particularly affected the results of the third doses, which began in July 2021, in the summer, when the pandemic wave was over. The follow-up of the unvaccinated, as well as that of the first doses, began in January 2021, in winter and during the second pandemic wave, which is when the risk of death from COVID-19 and that of all-cause deaths were significantly higher. This leads to underestimating the HR of all-cause deaths for individuals vaccinated with three or more doses during the follow-up period of this study.

There is also another common bias in studies of COVID-19 vaccine effectiveness, which can help explain the HR trend: the so-called case-counting window bias, which consists of considering those vaccinated in the 14 days following any inoculation as if they had not yet received the corresponding dose.

Regarding the case-counting window bias, we can cite a study [6] with data taken from the authorization trial of the Pfizer–BioNTech vaccine. It showed that an ineffective vaccine could appear effective at 48%, due to the above mentioned 14-day shift. In the first 14 days (from 1 to 15 January 2021) of Rosso’s trial, deaths were counted neither for the vaccinated nor for the unvaccinated individuals. For the remaining duration of the study, the deaths of individuals vaccinated in the first 14 days after the dose administration were not counted (as stated in the materials and methods section of the Rosso’s study). However, we cannot exclude that the deaths not counted for individuals vaccinated with one dose were attributed to the unvaccinated and, in cascade, the deaths of individuals vaccinated with two doses to those vaccinated with one dose, and so on.

Note that the Italian Superior Institute of Health (ISS), in an answer [18] to a specific question about the 14-day shift, stated the following:

“The Italian Superior Institute of Health, both in the scientific publications and in its reports published in the last two years, to evaluate the effectiveness of anti-COVID-19 vaccines, considers people who are diagnosed in the first 14 days after administration of the first dose, as “not vaccinated” (regardless of whether they have developed a serious disease or if they died).

There are mainly two reasons for this choice: 1. the protection of the vaccine requires approximately two weeks for the immune response to be developed against the virus; 2. the incubation period of the disease, i.e., the time from infection to the development of symptoms, varies from 2 to 14 days; it is emphasized that the diagnosis (in the pharmacy or in authorized laboratories) usually takes a few additional days. Therefore, a good part of the cases diagnosed within 14 days contracted the infection before the first dose was administered. In the case of evaluating adverse events from vaccines (e.g., anaphylactic shock) the choice to consider the date of administration of the vaccination as the moment of onset of exposure is clearly shared. In both cases, what has been done is in accordance with scientific knowledge and in line with what is suggested by national and international health bodies” [19].

In light of the ISS declaration reported above, we can assume that the health institutions in Pescara have conformed to the national indications. It would even appear that the authors of Rosso’s dataset have gone further. In fact, despite the follow-up starting 15 days after the vaccine administration, some deaths in the dataset (four for the first dose, one for the second, eight for the third) occurring within 14 days of the start of the follow-up (therefore within 28 days of administration of the dose) were assigned to the previous vaccination group.

However, even if the deaths had not been moved (apart from the few reported above) but only cancelled, the described effect would still exist, even if to a lesser extent.

Another bias likely influencing the results is the healthy-adherer bias, or healthy-vaccinee bias in the vaccination field. It is true that the priority was to vaccinate the so-called “fragile”. However, even before this obligation came into force, categories were also prioritized whose good health is an essential requirement, such as healthcare workers and the police, security, defense, and school personnel. In addition, the voluntary adhesion of the population not subject to obligations (direct or indirect, through the conditioning of the so-called green pass) can contribute to the aforementioned bias, as highlighted in the vast but little-known literature [20,21,22,23,24,25,26,27,28,29,30,31,32,33,34,35,36,37,38,39].

The healthy-adherer bias is much more powerful than commonly thought. Moreover, it is independent of the type of treatment one adheres voluntarily to, as it is also found in randomized controlled trials in placebo adherers (compared with in placebo non-adherers). It is more challenging to correct compared to the opposite effect of confounding by indication (subjects in worse health conditions are vaccinated first) [35], because the healthy-adherer bias can also be linked to features not captured by typical pharmaco-epidemiological databases, e.g., subjects more adherent to preventive therapies are often more likely to engage in behaviors consistent with a healthy lifestyle. These behaviors include maintaining a healthy diet, exercising regularly, moderating alcohol intake, avoiding illegal drugs or risky behaviors, seeking better quality health assistance, and having greater confidence in the benefits of a treatment, which can enhance a placebo effect. These unmeasured characteristics may be associated with mortality outcomes in observational studies. Accordingly, the healthy-vaccinee bias has shown huge effects in a national study linking mortality to COVID-19 vaccination status [38,39]. Indeed, it is plausible that, in observational studies, it also matters that the most fragile people, in the terminal stages of their diseases, could choose not to be vaccinated, or that their doctor does not think to vaccinate them (the so-called “frailty exclusion bias”).

The healthy-vaccinee bias likely continued to operate to varying degrees in 2022, throughout the follow-up of the analyzed study [9].

It is conceivable that the extent of the biases described above, although neither quantified nor quantifiable with sufficient approximation, go in the direction of increasing the disadvantage of the vaccinated compared to the unvaccinated.

Moreover, the third dose or booster was not foreseen at all, either by the authorization trials or by the vaccination protocols. It was introduced following the observation of the loss of the primary cycle vaccine effectiveness (VE), which was foremost highlighted in Israel, a state that had signed an agreement with Pfizer to monitor the VE on the general population. Therefore, even before confirmation by studies in other countries, in the summer of 2021 Pfizer requested an emergency authorization to the FDA for the third dose, then extended to other vaccines. This was a clear admission of the vaccines’ inability to guarantee a stable and long-lasting protection against COVID-19.

Another aspect to consider carefully comprises the HRs obtained for the covariates included in the model. The HRs of the covariates in the Cox proportional model represent, for the same vaccination doses, the comparison between subjects who have a specific comorbidity with subjects who do not have it. The results obtained from the model indicate that, for any dose, the HRs are significantly higher than the reference value. The unexpected result obtained in this analysis indicates that vaccinated subjects with at least one comorbidity have a higher risk of death from all causes than subjects vaccinated with the same doses, but without comorbidities. Considering these results, it appears necessary to make appropriate assessments regarding public health policies and re-evaluate the vaccination priority for specific categories of fragile subjects.

This study allows us to not only compare the most definitive outcome, death in the general population, in relation to vaccination status; but it also allows us to carry out a multivariate correction of a number of conditions and pathologies, substantially reducing the bias of confounding by indication.

### Limitations and Suggestions

However, this study also has some limitations. For example, the multivariate results for the second and third doses are to be considered as approximations. Indeed, despite the stratification, the Schoenfeld tests remained significant; we have therefore tried to overcome this limitation by calculating RSMT and RTML life expectancy indices.

A second limitation is that the results of this study have to be confirmed in other similar studies. Unfortunately, we are not aware of any other studies that have evaluated the total mortality in a population in association with individual COVID-19 vaccination status, simultaneously correcting for ITB and for confounding by indication. In fact, public Agencies such as the UK Office for National Statistics (ONS) have made all-cause mortality data by individual vaccination status available in England; but the ONS has not published datasets allowing for correction for factors other than age and sex. However, the importance of such information for public health policy would require such data to be collected and released systematically, to allow this type of study to be reproduced in different populations.

Moreover, despite the two-year follow-up in the Pescara study [9], confirmation of our estimate would require a longer observation. Therefore, we hope that further studies will extend the follow-up beyond what was already considered.

A third limitation is that the available information and the study design do not allow us to correct adequately for the four biases hypothesized above (harvesting effect, calendar-time bias, case-counting window bias, healthy-vaccinee bias), although it is reasonable to assume that none of the aforementioned biases would overturn the results of our study.

The study design to properly answer these questions could be a large pragmatic randomized controlled trial (RCT), with minimum exclusion criteria, long follow-up, and even longer observational extension. To overcome the ethical problems of not administering a COVID-19 vaccine to one of the randomized arms, or of administering it (out of a pandemic, emergency context), the solution could be to take advantage of widespread vaccine hesitancy. Indeed, after providing complete and balanced information based on the state of knowledge, a non-negligible percentage of the population remains unable to decide whether to be vaccinated or not. These persistently hesitant persons could be a valuable resource for scientific research. A voluntary opportunity could be offered to them: to participate in a well-designed RCT, thus contributing to a real advancement in scientific knowledge.

Further studies are certainly needed. However, despite the limitations described above, the results of this study can be an opportunity to rethink political choices about pandemic management and support greater caution in the future.

## 5. Conclusions

The correction of ITB has allowed us to eliminate remarkable distortions due to this bias from the original study about the effectiveness of COVID-19 vaccines, carried out in the province of Pescara, Italy. Moreover, the original study showed that the group who received at least a booster dose had an unlikely significantly lower risk of all-cause death versus the unvaccinated, unlike those vaccinated with one or two doses who had significantly higher risks than the unvaccinated.

We found all-cause death risks to be even higher for those vaccinated with one and two doses compared to the unvaccinated and that the booster doses were ineffective. We also found a slight but statistically significant loss of life expectancy for those vaccinated with 2 or 3/4 doses.

## Figures and Tables

**Table 1:** Characteristics of the sample.

	Unvaccinated ^a^	1 Dose ^b^	2 Dose ^c^	3/4 Dose ^d^
	(n = 290,727)	(n = 245,741)	(n = 234,287)	(n = 186,684)
Age in years (Mean, SD)	48.9 (20.8)	49.7 (20.7)	50.1 (20.7)	52.5 (20.2)
Gender (n,%)				
Females	148,770 (51.2)	127,121 (51.7)	121,516 (51.9)	97,440 (52.2)
Males	141,957 (48.8)	118,620 (48.3)	112,771 (48.1)	89,244 (47.8)
Risk factors and comorbidities (n,%)				
Hypertension	40,255 (13.8)	37,003 (15.1)	36,159 (15.4)	32,264 (17.3)
Diabetes	15,599 (5.4)	14,224 (5.8)	13,837 (5.9)	12,282 (6.6)
CVD	23,252 (8)	20,940 (8.5)	19,991 (8.5)	17,321 (9.3)
Kidney disease	5431 (1.9)	4718 (1.9)	4568 (1.9)	3793 (2)
Cancer	16,580 (5.7)	15,065 (6.1)	14,676 (6.3)	12,810 (6.9)
Infection	117,559 (40.4)	104,397 (42.5)	97,102 (41.4)	69,637 (37.3)
COPD	11,035 (3.8)	9802 (4)	9391 (4)	7683 (4.1)

SD = Standard deviation. ^a^ In this group were inserted never-vaccinated and vaccinated individuals before receiving one or more doses; ^b^ in this group were inserted all individuals vaccinated with 1 dose and all individuals vaccinated before receiving 2 or more doses; ^c^ in this group were inserted all individuals vaccinated with 2 doses and all individuals vaccinated before receiving 2 or more doses; ^d^ in this group were inserted all individuals vaccinated with 3 or more doses.

**Table 2:** COVID-19-related death classifications.

	Unvaccinated	1 Dose	2 Doses	3/4 Doses	Total Sample
Total COVID-19-related deaths, n (%) ^1^	573 (28.9)	66 (20.1)	225 (11.5)	658 (25.8)	1522 (22.3)
Deaths without severe COVID-19, n (%) ^2^	78 (13.6)	34 (51.5)	68 (30.2)	267 (40.6)	447 (29.4)
Same day for swab and death, n (%) ^2^	18 (3.1)	0 (0)	8 (3.6)	22(3.3)	48 (3.2)
Deaths > 90 days from swab, n (%) ^2^	107 (18.7)	43 (65.2)	83 (36.9)	244 (37.1)	477 (31.3)
Cumulative questionable classifications, n (%) ^2^	142 (24.8)	47 (71.2)	100 (44.4)	330 (50.2)	619 (40.7)

^1^ Percentage compared to all-cause death; ^2^ percentage compared to total COVID-19-related death.

**Table 3:** All-cause deaths and hazard ratios (HRs) according to vaccination status in univariate and multivariate analyses.

Covariate	1 Dose		2 Doses		3/4 Doses	
	Univariate	Multivariate	Univariate	Multivariate	Univariate	Multivariate
	HR (95% CI)	HR (95% CI)		HR (95% CI)		HR (95% CI)
Groups	0.88 (0.78–1.00) ^‡^	2.40 (2.00–2.88) **	1.23 (1.16–1.32) *	1.98 (1.75–2.24) **	1.21 (1.14–1.29) *	0.99 (0.90–1.09)
Hypertension	12.59 (11.58–13.69) *	1.49 (1.23–1.82) **	11.47 (10.76–12.23) *	/	9.65 (9.09–10.24) *	1.24 (1.11–1.39) **
Diabetes	8.07 (7.31–8.90) *	2.00 (1.60–2.49) **	6.71 (6.23–7.23) *	1.74 (1.38–2.20) **	5.90 (5.51–6.31) *	1.68 (1.48–1.90) **
CVD	11.56 (10.63–12.57) *	1.60 (1.31–1.96) **	10.88 (10.21–11.60) *	1.78 (1.44–2.20) **	10.03 (9.45–10.63) *	1.86 (1.65–2.09) **
Kidney disease	17.89 (16.08–19.90) *	1.77 (1.35–2.34) **	16.83 (15.56–18.20) *	2.44 (1.84–3.24) **	15.89 (14.78–17.08) *	2.47 (2.11–2.89) **
Cancer	9.34 (8.51–10.25) *	/	8.65 (8.07–9.27) *	/	7.62 (7.15–8.12) *	/
Infection	0.58 (0.53–0.63) *	/	0.35 (0.32–0.38) *	/	0.61 (0.58–0.66) *	/
Age	1.11 (1.11–1.11) *	/	1.11 (1.11–1.12) *	/	1.12 (1.11–1.12) *	/
Sex	0.87 (0.81–0.95) *	1.50 (1.27–1.78) **	0.95 (0.89–1.01)	/	0.98 (0.93–1.04)	1.37 (1.24–1.51) **
COPD	7.11 (6.40–7.91) *	2.01 (1.56–2.60) **	6.28 (5.79–6.82) *	2.89 (2.18–3.84) **	5.96 (5.52–6.43) *	1.85 (1.59–2.15) **

HRs = hazard ratios; CI = Confidence Interval; ^‡^ *p*-value = 0.044; * significance with *p*-value ≤ 0.001; ** significance with *p*-value < 0.0001. The HRs indicated with “/” are the covariate stratified in order to correct the assumptions of the Proportional Cox Mode.

**Table 4:** Estimate of Restricted Mean Survival Time and between-group contrast in 2 doses versus unvaccinated.

Restricted Mean Survival Time (RMTS) (τ = 739 Days)				
Groups	Estimate	SE	95% CI	
RMST two doses (arm1)	728.9	0.3	728.3–729.5	
RMST unvaccinated (arm0)	731.6	0.2	731.3–731.9	
Restricted Mean Time Lost (RMTL)				
RMTL two doses (arm1)	10.1	0.3	9.5–10.7	
RMTL unvaccinated (arm0)	7.4	0.2	7.0–7.7	
Between-group contrast				*p*-value
RMST (arm1-arm0)	−2.7		−3.4–−2.0	<0.0001
RMTL (arm1/arm0)	1.37		1.27–1.48	<0.0001

**Table 5:** Estimate of Restricted Mean Survival Time and between-group contrast in 3 doses versus unvaccinated.

Restricted Mean Survival Time (RMTS) (τ = 579 Days)				
Groups	Estimate	SE	95% CI	
RMST three doses (arm1)	573.7	0.1	573.5–573.9	
RMST unvaccinated (arm0)	574.	0.1	574.2–574.7	
Restricted Mean Time Lost (RMTL)				
RMTL three doses (arm1)	5.3	0.1	5.1–5.5	
RMTL unvaccinated (arm0)	4.6	0.1	4.3–4.8	
Between-group contrast				*p*-value
RMST (arm1-arm0)	−0.8		−1.1–−0.5	<0.0001
RMTL (arm1/arm0)	1.17		1.10–1.24	<0.0001

## Data Availability

The raw data supporting the conclusions of this article will be made available by the authors on request.
